# Do Progestin-Only Contraceptives Contribute to the Risk of Developing Depression as Implied by Beta-Arrestin 1 Levels in Leukocytes? A Pilot Study

**DOI:** 10.3390/ijerph15091966

**Published:** 2018-09-09

**Authors:** Keisha Smith, Sanket Nayyar, Tanu Rana, Anthony E. Archibong, Kimberly R. Looney, Tultul Nayyar

**Affiliations:** Department of Neuroscience & Pharmacology, Meharry Medical College, 1005 Dr. D. B. Todd Jr. Blvd, Nashville, TN 37208, USA; ksmith12@email.mmc.edu (K.S.); snayyar17@email.mmc.edu (S.N.); trana@mmc.edu (T.R.); aarchibong@mmc.edu (A.E.A.); klooney@mmc.edu (K.R.L.)

**Keywords:** beta-arrestin 1, depression, combination-oral contraceptives, progestin-only contraceptives, mononuclear leukocytes, women

## Abstract

We reported previously that reduction in beta-arrestin 1 (β-AR 1) protein levels in peripheral blood mononuclear leukocytes (PBMC) significantly correlated with the severity of depressive symptoms in reproductive women. In this pilot study, we used β-AR 1 protein levels in PBMC as a marker for developing depressive symptoms and the Hamilton Depression Rating Scale (HAM-D) scores to assess potential mood-related side effects of oral contraceptive use for routine birth control among women. We evaluated 29 women in this study. We enrolled the participants in three groups: Estrogen-progestin combination-oral contraceptives (COC, *n* = 10), progestin-only contraceptives (POC, *n* = 12), and non-hormonal or no contraceptives (NC, *n* = 7). We determined the β-AR 1 protein levels in PBMCs by enzyme-linked immunosorbent assay (ELISA). We found that women in the POC group had significantly higher HAM-D scores compared to those in the COC (*p* < 0.0004) and NC (*p* < 0.004). The levels of β-AR 1 protein were significantly attenuated in women in the POC group compared to women in the NC group (*p* = 0.03). Our findings suggest that the use of POC is a potential risk factor for developing depressive symptoms.

## 1. Introduction

Depression and anxiety, among the most prevalent and disabling chronic diseases affecting reproductive-aged women worldwide, can contribute to adverse reproductive health outcomes. Women across different populations are twice as likely to experience depression as men during their reproductive years [1]. Depression and anxiety disorders also often go undetected and untreated among poor, unemployed, and less educated women [2]. Additionally, since contraceptive agents are widely used by reproductive women, it is important to know if any association exists between contraceptive use and depressive symptoms. It has been suggested in a review article that modern contraceptives with a lower-dosage of steroids do not have a clinically relevant impact on women’s mood, as compared to higher doses of steroids that were used during the 1970’s when depression was the known side effects of oral contraceptives use [3]. However, a long-term study on more than a million women in Denmark, with no history of depression for 13 years, found that those who used hormonal contraceptives had a fifty percent greater risk of developing depression within six months of using the contraceptives than those who did not use it [4].

Additional studies have delivered an ambiguous pattern of results. One study [5] found no association between oral contraceptive use and depressive symptoms, and others suggested [6,7] that hormonal contraception was associated with better mood. Few other studies reported the development of depressive symptoms are a known reason for cessation of hormonal contraceptive use [8,9,10]. It was also found that non-hormonal or no contraceptive (NC) use (i.e., condoms and withdrawal vs. COCs and long-acting reversible contraception) to be associated with higher depressive and stress symptoms [11]. From this, it is evident that more studies are needed, in particular, to determine whether relationships differ according to the type of contraceptive agent used.

Currently, there are many different types of oral contraceptives available. Combination oral contraceptives containing both estrogen and progestin (COC) and progestin-only contraceptives (POC) are the two major types with so many variations in the composition of the components. Studies on COC and POC have yielded different results. A double-blind randomized placebo-controlled trial did not find an effect of COC on depressed mood [12], but progestins such as medroxyprogesterone acetate (MPA) in Depo-Provera [13] and levonorgestrel (LNG) in Mirena [4] have been reported to be associated with an increase in depressive symptom manifestation. Additional data suggests a protective effect of COC and a deleterious effect of POC with regards to mood disorders [14]. Moreover, a case study on a patient exhibited signs of self-mutilation and depressive symptoms three and a half weeks after using Depo-Provera; this patient did not have any significant personal or family psychiatric history [15]. Interestingly, a recent analysis of a retrospective study concluded that women using POC did not appear to experience more depressive symptoms or mood changes than women on other hormonal contraceptives, and that they may experience milder bouts of depression than women who did not use contraception [16]. Due to these contradicting findings, additional studies are warranted to determine the relationship between hormonal contraceptives and depressive symptoms, and whether such a relationship is dependent on the type of contraceptive used.

We present beta-arrestin 1 (β-AR 1) protein level, a novel analyte in peripheral blood mononuclear leukocytes (PBMCs), which can be used as an early marker or may have mediated the observed changes in mood. The approach offers the scientific basis for a possible contraceptive side effect that has not been well-explained previously. The β-AR proteins, which regulate the G protein receptor coupling, play an important role in the pathophysiology of mood disorders and in the mechanisms underlying the effects of antidepressants [17,18,19,20]. The β-AR 1 protein and mRNA levels in PBMCs of patients with untreated major depression were significantly lower than the levels found in healthy subjects and their levels also correlated significantly with the severity of the depressive symptoms [17,18]. Treatment with antidepressants elevated the β-AR 1 protein and mRNA levels [17,19]. We [21], along with others [17,22], proposed the β-AR 1 levels in PBMCs as a biochemical marker for depressive symptoms in humans. We reported that β-AR 1 protein levels were significantly lower in women with premenstrual dysphoric disorder (PMDD), a severe form of premenstrual syndrome (PMS), compared to those women with the non-severe form of PMS. Additionally, the reduction in β-AR 1 levels correlated with the increase in core symptoms of depression on the Hamilton Depression Rating Scale (HAM-D) [21]. Findings from these reports suggest that the assessment of β-AR-1 protein levels may prove useful for diagnosing depressive symptoms with high sensitivity and specificity [23].

Therefore, to assess potential mood-related side effects of oral contraceptives, we measured the β-AR-1 protein levels in PBMCs and recorded the HAM-D scores for women in the NC, COC, and POC groups on a single day when they were between days 21–25 of the menstrual cycle, the susceptible period for PMS/PMDD. This period was selected because it has previously been suggested that women with PMS are more likely to suffer from depressive symptoms during COC use [24], and another study reported that certain combinations of progestogens are less suitable for women with PMS [25].

## 2. Materials and Methods

### 2.1. Participant Recruitment

Participant and Clinical Interaction Resource (PCIR) at the Meharry Clinical Research Center helped us recruit the participants for this study by putting up flyers, posting advertisements in publications approved by Meharry, and through recommendations from existing participants in similar studies. Inclusion criteria were as follows: (1) Women between 18–42 years of age who used either hormonal (estrogen-progestin combination agents, progestin-only agents) or non-hormonal contraceptives, and those in the same age group who did not use contraceptives for at least three months before enrollment in the study; (2) in good general health with no clinically significant systemic abnormalities; (3) did not suffer from chronic inflammatory diseases; (4) had not been treated with antidepressants within the last four weeks before the study; and (5) able to understand, read, and speak English, as well as understand the procedures and use/disclosure of protected health information (PHI) and subsequently give consent. Exclusion criteria were as follows: (1) history or evidence of clinically significant physical disorders; (2) existence or history of clinically significant and diagnosable major psychiatric disorders and/or chronic inflammatory diseases, as well as past diagnoses of schizophrenia, bipolar disorder, or primary anxiety disorder; and (3) any antidepressant/psychotropic drug or substance use within the past four weeks other than caffeine, and nicotine. Women using hormonal contraceptives for treatment or prophylaxis of gynecological (for e.g., heavy menstrual bleeding) or dermatological (for e.g., acne) conditions were also excluded.

Women interested in the study were given a complete description of the study. Written informed consent was obtained from the participants for a 24 mL blood donation. The women were evaluated using the Mini-International Neuropsychiatric Interview [26]. The severity of depression was determined using the 17-item HAM-D questionnaire. Participants with scores of >19 were considered depressed. The participants completed the HAM-D questionnaire before donating blood. The HAM-D scores and blood samples were collected when the women were between days 21–25 into their menstrual cycle. The participants were enrolled in the following groups: Estrogen-progestin combination-oral contraceptives (COC), progestin-only contraceptives (POC), and non-hormonal or no-contraceptives (NC). The NC group consisted of women with a regular menstrual cycle (25–33 days), who reported no use of hormonal contraceptives for at least three months prior in order to participate in this study. The study was approved by the Institutional Review Board at Meharry Medical College under the protocol number 15-03-364 (02/2018).

### 2.2. HAM-D Scores

The HAM-D score of each woman was calculated based on her answers to the HAM-D questionnaire as previously described [21] (where 0 = absent). Briefly, depressed mood (0–4), difficulty in work activities (0–4), agitation (0–2), insomnia early (difficulty in falling asleep) (0–2), insomnia middle (complains of being restless and disturbed during the night) (0–2), insomnia late (waking in early hours of the morning and unable to fall asleep again) (0–2), psychological anxiety (0–4), somatic anxiety (gastrointestinal, indigestion, cardiovascular, palpitation, headaches, respiratory, and genitourinary, etc.) (0–4), and somatic symptoms (loss of appetite, heavy feeling in abdomen, and constipation) (0–2) [27].

### 2.3. Isolation of Mononuclear Leukocytes

Participant identities and group assignments for all blood samples were blinded upon collection using anonymized coding. Biochemical analyses were conducted by laboratory personnel who had no involvement or knowledge of the details of the sample collection. Vacutainer cell preparation tubes with sodium citrate (CPT) (BD Biosciences, San Jose, CA, USA) were used to collect 24 mL of blood from each participant using the venipuncture technique. Isolation of mononuclear leukocytes was performed according to the manufacturer’s instructions. Briefly, CPT tubes were inverted gently to mix the blood with the anticoagulant additive and centrifuged at 1500× *g* at room temperature (18–25 °C) for a minimum of 20 min. The mononuclear cells (the white buffy coat underneath the plasma layer) were collected into a 15-mL conical tube, washed with phosphate buffered saline (PBS), and centrifuged at 400× *g* for 15 min. The supernatant was discarded. The samples were washed again with PBS and centrifuged at 400× *g* for 10 min. The cell pellets were stored at −80 °C for later use.

### 2.4. Enzyme-Linked Immunosorbent Assay (ELISA)

The protein was extracted from the PBMC samples by using 200 μL of Pierce RIPA Buffer (Pierce BioTechnology; Rockville, IL, USA product #89900) containing Halt protease inhibitor cocktail (Pierce BioTechnology; Rockville, IL, USA product #783430). The mixture was centrifuged at a maximum speed for 15 min. The supernatant was collected and stored at −80 °C for ELISA. The ELISA protocol from the manufacturer (Human Arrestin β1:ARRβ1 ELISA kit; MyBioSource, San Diego, CA, USA, catalog # MBS725731) was used to determine the levels of β-AR 1 protein. All assays were done in duplicates and measurements were obtained using the xMark microplate spectrophotometer (Bio-Rad Laboratories, Inc., Hercules, CA, USA). Briefly, 10 μL of balance solution and 50 μL of the conjugate were added to each well, except the blank control well. The plate was mixed using a plate shaker and incubated for 1 h at 37 °C. Next, the mixture was removed and the plate was washed five times with 1× wash solution. Then, 50 μL of substrate A and 50 μL substrate B were added to each well. The plate was covered and incubated for 15 min at 37 °C. Finally, 50 μL of stop solution was added to each well and the plate was read at 450 nm. The β-AR 1 protein level in each sample was determined using a standard curve constructed for each assay. The protein concentrations of each sample was determined using the BCA assay (Pierce BioTechnology; Rockville, IL, USA, product #23225). The β-AR 1 protein level in each sample was then calculated based on the total protein content of each sample to obtain the β-AR 1 level as ng per mg of protein. The standard curve and the % CV for the duplicate samples were all within the acceptable range for each assay.

### 2.5. Statistical Analyses

Results are expressed as mean ± SEM unless otherwise indicated. The GraphPad Prism software (Graph-Pad Software, Inc., San Diego, CA, USA) was used for all the statistical analysis. Results with *p* < 0.05 were considered statistically significant. Data were analyzed by ANOVA using the contraceptive type as a factor in three levels, followed by post-hoc analyses using Bonferroni’s multiple comparisons when a significant effect was revealed by ANOVA. Regression analyses were conducted between the type of contraceptive and the β-AR 1 protein levels. Potential confounders (race, age, body weight, smoking behavior, and the day of the menstrual cycle when blood samples and HAM-D scores were collected) were also assessed.

## 3. Results

### 3.1. Demographics

A total of 29 women (NC = 7, COC = 10, POC = 12) participated in this study. Participant characteristics are listed in Table 1.

The body weights of women in the POC group were significantly lower than those of women in the NC group (*p* < 0.05). There was no significant difference in body weights between women in the NC group and those in the COC group (*p* = 0.19), and between women in the COC group and those in the POC group (*p* = 0.75).

### 3.2. HAM-D Scores

**Figure 1:**
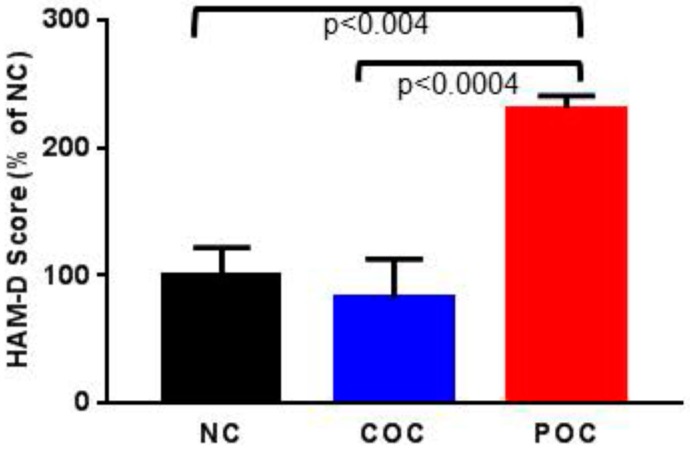
The HAM-D scores (mean ± SEM) among women using non-hormonal or no contraceptives (NC), estrogen-progestin combination-oral contraceptives (COC), and progestin-only contraceptives (POC) were compared. Data are expressed as percentages of values for women in the NC group. Women in the POC group scored significantly higher on HAM-D than women in the NC group (*p* < 0.004) or women in the COC group (*p* < 0.0004). There was no significant difference in the HAM-D score between NC and COC women (*p* = 0.42).

Based on the participants’ HAM-D scores, we found that out of 29 women, 23 (79%) women were not depressed (HAM-D < 19; 8.0 ± 1.09) and six (21%) were depressed (HAM-D > 19; 20.22 ± 1.3). The body weight of not depressed (202 ± 13.37) vs. depressed (160.4 ± 14.28) women were not statistically significant (*p* = 0.19). All the six depressed women belonged to the POC group. The group-wise analysis of HAM-D scores are as follows: NC, (7.42 ± 1.63, CI 95% 3.43–11.42); COC, (6.1 ± 1.86, CI 95% 1.88–10.32); and POC, (17.08 ± 1.82, CI 95% 13.07–21.1). ANOVA revealed that the types of contraceptives used significantly affected the HAM-D scores (F [2,26] = 11.64, *p* = 0.0002). Post-hoc analyses revealed that women in the POC group had significantly higher HAM-D scores than those in the COC group (*p* < 0.0004) and the NC group (*p* < 0.004) (Figure 1). 

ANOVA assessing the core symptoms of depression on the HAM-D scale demonstrated significant differences in the following parameters: Insomnia early, (F [2,21] = 3.88, *p* = 0.03); insomnia middle, (F [2,21] = 6.55, *p* = 0.006); psychological anxiety, (F [2,21] = 6.12, *p* = 0.008); somatic anxiety, (F [2,21] = 4.16, *p* = 0.03); and somatic symptoms, (F [2,21] = 5.16, *p* = 0.01). Post-hoc analysis revealed that women in the POC group had significantly higher scores for the following characteristics: Insomnia early (*p* = 0.04), insomnia middle (*p* = 0.01), psychological anxiety (*p* = 0.02), and somatic symptoms (*p* = 0.02) compared to women in the NC group. Women in the POC group also had significantly higher scores for insomnia middle (*p* = 0.02) and psychological anxiety (*p* = 0.01), than women in the COC group. However, post-hoc tests did not reveal any significant difference in somatic anxiety among the three groups. There was also no significant difference in the scores for any of the core symptoms of depression between women in the NC group and those in the COC group (Table 2).

### 3.3. β-AR 1 Protein Levels

**Figure 2:**
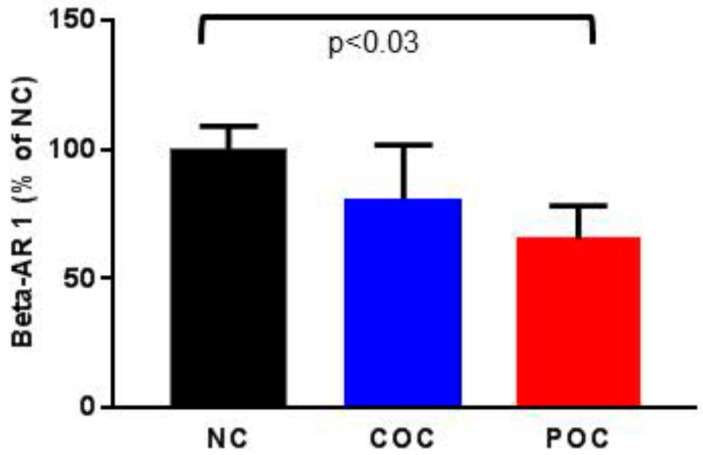
The β-arrestin 1 protein levels (mean ± SEM) in the leukocytes were compared among women using non-hormonal or no contraceptives (NC), estrogen-progestin combination-oral contraceptives (COC), and progestin-only contraceptives (POC). Data are expressed as percentages of values for women in the NC group. The β-arrestin1 (ng/mg) levels were significantly attenuated in women in the POC group, compared to women in the NC group (*p* < 0.03). There were no statistically significant differences in the β-AR 1 levels between women in the NC group and those in the COC group, and between women in the COC group and those in the POC group.

The β-AR 1 protein levels in PBMCs were as follows: NC, 4.44 ± 0.40; COC, 3.57 ± 0.76; and POC, 2.87 ± 0.39. Though our data seems to imply a downward trend in β-AR 1 levels, i.e., NC > COC > POC, ANOVA did not reveal any significant effect of contraceptive types on β-AR 1 levels. Nonetheless, regression analysis demonstrated that β-AR 1 protein levels in women in the POC group were significantly attenuated compared to those in women in the NC group (*p* = 0.03). There was no statistically significant difference in β-AR 1 protein levels between women in the NC group and those in the COC group (*p* = 0.81), and between women in the COC group and those in the POC group (*p* = 0.052) (Figure 2). 

Table 3 compares the β-AR 1 levels with the independently obtained HAM-D scores among women in the NC, COC, and POC groups. We found that women in the POC group had significantly higher HAM-D scores compared to those in the NC group and COC group. Interestingly, women in the POC group also had lower levels of β-AR 1 protein than women in the NC group.

### 3.4. Potential Confounders

We also assessed potential confounders (race, age, body weight, smoking behavior, number of pregnancy, number of children, and the day of the menstrual cycle when blood samples and HAM-D scores were collected) that could affect the HAM-D scores and β-AR 1 protein levels. We found that none of these factors yielded significant effects on either the HAM-D scores or β-AR 1 protein levels (data not shown).

## 4. Discussion

This study offers a scientific basis for determining possible mood-related side effects of oral contraceptives that have not been explored previously. From a group of 29 participating women, we found that women in the POC group had attenuated β-AR 1 protein levels compared to women in the NC group. We and others have demonstrated previously that reduction in the β-AR 1 protein levels significantly correlated with the development of depressive symptoms [17,18,21].

Our HAM-D score data revealed that six out of the 12 women in the POC group were depressed (HAM-D > 19). In contrast, participants in the COC and NC groups were not depressed. Among the several core symptoms of depression on the HAM-D scale, scores for insomnia (early and middle), somatic symptoms, and psychological activity were significantly higher among women in the POC group compared to those in the NC group. Furthermore, scores for insomnia (middle) and psychological activity were also significantly higher among women in the POC group than women in the COC group (Table 2). These findings are in line with previous studies reporting an increase in depressive symptoms associated with the use of POC [13,28,29]. Our data on β-AR 1 protein levels revealed that women in the POC group exhibited a significant reduction in β-AR 1 levels compared to women in the NC group (Figure 2). We and others have previously shown the reduction in β-AR 1 levels to be significantly correlated with the HAM-D scores [17,18,21]. In this study, we found that women in the POC group had higher HAM-D scores and showed a reduction in β-AR 1 protein levels compared to women in the COC group, although the difference in β-AR 1 protein levels is not statistically significant (Table 3). We also found that women in the COC group had HAM-D scores and β-AR 1 levels that were similar to those for women in the NC group (Table 3). Therefore, we postulate that the use of POC may be a potential risk factor for developing depressive symptoms. Since β-AR 1 has been shown to facilitate estrogen-mediated neuroprotection [30], it is possible that the estrogen in COC attenuates a progestin’s effect on β-AR 1 levels.

There are some plausible mechanisms that may explain the relationship between the use of contraceptives and mood status. Ovarian hormones have been shown to play a role in serotonin neurotransmission [31,32,33], which is the most relevant pathophysiological correlate of depression [34]. Serotonin neurotransmission involves G-protein-coupled receptor (GPCR) signaling [35], in which β-AR 1 plays a pivotal role [18]. Progestins, which are a type of synthetic progesterone, not only mimic the actions of endogenous progesterone, but also have divergent biological effects with respect to endogenous progesterone. Preclinical studies have shown that progesterone and progestins can modulate brain functions through differential binding and the activation of progesterone receptors, activation of various intracellular pathways, and differential modulation of the synthesis and release of several neurotransmitters including serotonin [36,37]. Adverse mood effects induced by POC may also be due to the action of progesterone metabolites on the γ-aminobutyric acid receptor complex [38], which has a documented role in mood regulation [39]. External progestins increase levels of monoamine oxidase, possibly more effectively than endogenous progesterone does; hence, progestins can potentially degrade serotonin more rapidly and thus enhances the symptoms of depression and irritability [40]. Estrogens are shown to increase serotonin levels [41] and have an antidepressant-like effect on neurotransmitters such as those present in the dopaminergic, catecholaminergic, and GABAergic neurons [42]. Therefore, it is possible that the estrogen in the COC reduces the risk of developing depressive symptoms among women who use COC [43].

Several limitations exist for this study, including a small sample size, selection bias, and a demographic imbalance. This study did not analyze the following factors: The length of time hormonal contraceptives were used, data on miscarriages and abortions, assessment of life events, and psychosocial factors [44,45]. The categories of contraception listed (NC, COC, and POC products) are quite heterogeneous, with individual methods within those categories having different effects and mechanisms of action. With POCs in particular, DMPA (higher serum levels of progestin) vs. progestin-only pills or IUDs (lower serum levels of progestin) have different effects on brain chemistry, and several different progestins combined with the varying amount of estrogen component in COCs act differently on mood symptoms [46].

## 5. Conclusions

Safe and effective methods of contraception represent a critical component of preventive health care. The implications derived from our findings show an association between POC and depressive symptoms, in addition to β-AR 1 protein levels in PBMC, as a possible biomarker and mechanism mediating the observed association. In conclusion, this pilot study points to the use of POC as a potential risk factor for developing depressive symptoms. It can be assumed that not all depressive symptoms experienced by POC users are drug-related. In future studies, a larger number of subjects should preferably be followed prospectively for HAM-D scores and β-AR 1 protein levels before the start of COC or POC agent use. With such a study design, it would be possible to identify subjects who develop depressive symptoms due to COC or POC use, and will be able to confirm the causal relationship between developing depressive symptoms and the type of contraceptive agent use. While most women will not experience clinical depression soon after starting hormonal contraceptives, doctors should be on the lookout for potential changes in mood in the first few months. Additionally, women starting on any kind of hormonal contraceptive should be careful to note any changes—good or bad, physical or mental—and discuss them with a doctor.

## Figures and Tables

**Table 1:** Participant characteristics.

Women (*n*) = 29	NC (*n* = 7, 24%)	COC (*n* = 10, 34%)	POC (*n* = 12, 41%)
Black	5	8	12
White	2	2	-
Age (Average, Range)	31 (32–38)	28 (24–36)	29 (19–38)
Weight (Average, Range)	237 (170–360)	188 (126–240)	173 (116–254)
NC vs. POC, *p* = 0.05 *			
NC vs. COC, *p* = 0.19			
COC vs. POC, *p* = 0.75			
Smoker	3	-	2
Nonsmoker	4	10	10
Pregnancies (Average, Range)	2 (0–5)	0 (0–1)	2 (0–5)
Children # (Average, Range)	2 (0–4)	0	2 (0–4)
Contraceptives (Name, Number)	Essure^®^ 1	Orthro Tri-Cyclen^®^ 3	Mini-Pill (Norethindrone) 2
	Paragard^®^ 1	Necon 1/35^®^ 2	IUD (Mirena^®^) 4
	No contraceptive 5	Junel Fe 1/20^®^ 2	IUD (Kyleena^®^) 1
		Generic Yaz 1	Implant (Nexplanon^®^) 4
		Lo Loestrin Fe^®^ 2	Injection (Depo Provera^®^) 1

NC, non-hormonal or no contraceptives; COC, estrogen-progestin combination-oral contraceptives; POC, progestin-only contraceptives; IUDs, Intrauterine devices; * Significantly different.

**Table 2:** Core symptoms of depression on the HAM-D scale (mean ± SEM).

Core Symptoms	NC	COC	POC	NC/COC (*p* Value)	NC/POC *p* Value)	COC/POC (*p* Value)
Depressed mood	1.0 ± 0.25	0.88 ± 0.35	2.0 ± 0.37	>0.99	0.12	0.07
Work & activities	0.83 ± 0.4	0.44 ± 0.17	1.44 ± 0.37	>0.99	0.64	0.08
Feelings of guilt	0.33 ± 0.21	0.33 ± 0.23	1.2 ± 0.32	>0.99	0.12	0.07
Agitation	0.5 ± 0.34	0.5 ± 0.33	1.0 ± 0.23	>0.99	0.85	0.86
Early insomnia	0.16 ± 0.16	0.44 ± 0.17	1.11 ± 0.3	>0.99	0.04 *	0.15
Middle insomnia	0.16 ± 0.16	0.33 ± 0.16	1.2 ± 0.27	>0.99	0.01 *	0.02 *
Late insomnia	0.16 ± 0.16	0.55 ± 0.24	0.77 ± 0.22	0.78	0.25	>0.99
Psychological anxiety	0.33 ± 0.21	0.44 ± 0.24	1.6 ± 0.37	>0.99	0.02 *	0.01 *
Somatic anxiety	0.66 ± 0.33	0.77 ± 0.32	2.0 ± 0.4	>0.99	0.07	0.06
Somatic symptoms	>0.99	0.22 ± 0.14	0.77 ± 0.22	>0.99	0.02 *	0.07

NC, non-hormonal or no contraceptives; COC, estrogen-progestin combination-oral contraceptives; and POC, progestin-only contraceptives. * Statistically significant (*p* < 0.05).

**Table 3:** Comparison of β-AR 1 protein levels (ng/mg) with independently obtained HAM-D score among women in the NC, COC, and POC groups.

Outcomes	NC	COC	POC
β-AR 1 (Mean ± SEM)	4.44 ± 0.40	3.57 ± 0.76	2.87 ± 0.39
NC vs. POC, *p* = 0.03 *			
NC vs. COC, *p* = 0.81			
COC vs. POC, *p* = 0.052			
HAM-D (Mean ± SEM)	6.0 ± 1.3	6.4 ± 2.04	17.6 ± 2.15
NC vs. POC, *p* = 0.004 **			
NC vs. COC, *p* = 0.42			
COC vs. POC, *p* = 0.0004 ***			

NC, non-hormonal or no contraceptives; COC, estrogen-progestin combination-oral contraceptives; POC, progestin-only contraceptives; β-AR 1, β-arrestin 1; HAM-D, Hamilton Depression Rating Scale; and NS, not significant. * *p* < 0.05, ** *p* < 0.005, *** *p* < 0.0005.
